# Splenectomy Alters Distribution and Turnover but not Numbers or Protective Capacity of *de novo* Generated Memory CD8 T-Cells

**DOI:** 10.3389/fimmu.2014.00568

**Published:** 2014-11-06

**Authors:** Marie T. Kim, John T. Harty

**Affiliations:** ^1^Interdisciplinary Program in Immunology, University of Iowa, Iowa City, IA, USA; ^2^Department of Microbiology, University of Iowa, Iowa City, IA, USA; ^3^Department of Pathology, University of Iowa, Iowa City, IA, USA

**Keywords:** splenectomy, memory, CD8 T-cells, homeostatic proliferation

## Abstract

The spleen is a highly compartmentalized lymphoid organ that allows for efficient antigen presentation and activation of immune responses. Additionally, the spleen itself functions to remove senescent red blood cells, filter bacteria, and sequester platelets. Splenectomy, commonly performed after blunt force trauma or splenomegaly, has been shown to increase risk of certain bacterial and parasitic infections years after removal of the spleen. Although previous studies report defects in memory B-cells and IgM titers in splenectomized patients, the effect of splenectomy on CD8 T-cell responses and memory CD8 T-cell function remains ill defined. Using TCR-transgenic P14 cells, we demonstrate that homeostatic proliferation and representation of pathogen-specific memory CD8 T-cells in the blood are enhanced in splenectomized compared to sham surgery mice. Surprisingly, despite the enhanced turnover, splx mice displayed no changes in total memory CD8 T-cell numbers nor impaired protection against lethal dose challenge with *Listeria monocytogenes*. Thus, our data suggest that memory CD8 T-cell maintenance and function remain intact in the absence of the spleen.

## Introduction

The spleen plays a key role in optimizing immune responses to pathogens. As the largest secondary lymphoid organ, the spleen allows for efficient antigen presentation by APCs to B- and T-cells, and has been suggested to regulate antigen tolerance, among other aspects of the immune system (1, 2). The spleen contains major types of mononuclear cells, such as DCs that provide signals to optimally activate T-cells (1). Removal of this organ can result in severe infections that can occur long after splenectomy is performed (3–5). Due to overwhelming post-splenectomy infection (OPSI) from bacteria, such as *Streptococcus pneumoniae, Haemophilus infleunzae*, and *Neisseria meningitis*, there have been multiple studies on the defects of B-cells and antibody production (6–8). Although many of the encapsulated-bacterial infections of splenectomized (splx) patients can be attributed to defects in IgM memory B-cells, there have also been reports of more severe viral infections (9) and a higher relative risk for parasitic infection post-splenectomy (10–15). Investigating possible defects in T-cell immunity may grant insight into why splx individuals have increased susceptibility or worse symptoms with viral or parasitic infections.

Many studies of impaired T-cell responses post-splenectomy have been performed *in vitro* on lymphocytes isolated from whole blood of splx patients at intermittent timepoints (11, 12). The effect of splenectomy on CD8 T-cell responses and memory CD8 T-cells remains incompletely defined (14, 16). Here, we address this knowledge gap through comprehensive analysis of CD8 T-cell kinetics, distribution, phenotype, and protective capacity in sham and splx mice induced by lymphocytic choriomeningitis virus (LCMV)-Armstrong.

## Materials and Methods

### Mice and infection

Age-matched female C57BL/6 (Thy1.2), splx, and sham-treated mice were acquired from the National Cancer Institute (NCI; Bethesda, MD, USA) or Jackson Laboratory (Bar Harbor, ME, USA). Thy1.1+ P14, TCR-transgenic mice specific for LCMV gp33–41, have been previously described (17) and maintained by sibling × sibling mating. All mice were housed under pathogen-free conditions and used for experiments at 6–10 weeks of age. After infection, mice were transferred to BSL2 housing. All animal studies and procedures were approved by the University of Iowa Animal Care and Use Committee, under PHS assurance, Office of Laboratory Animal Welfare guidelines. The model pathogen – LCMV-Armstrong (2 × 10^5^ PFU/mouse) – was the primary pathogen utilized in these studies. Challenge experiments were performed with virulent *Listeria monocytogenes*, expressing the GP33-41 epitope of LCMV (XFL203) (18) at 1 × 10^6^ CFU/mouse i.v.

### Memory CD8 T-cell generation and protection assay

To generate memory P14 CD8 T-cells, 2 × 10^4^ naïve Thy1.1 P14 CD8 T-cells were transferred into naïve Thy1.2 B6 hosts that were subsequently infected with LCMV-Armstrong i.p. For protection studies, D99 memory sham and splx mice were challenged with virulent *L. monocytogenes* (XFL203) intravenously. Three days post-challenge, mice were sacrificed and CFU of *L. monocytogenes* were quantified per gram of liver.

### Antigen detection assay and BrdU incorporation

Naïve B6 mice were infected with 2 × 10^5^ PFU LCMV-Armstrong i.p. on D-15 and D-6. At D0, naïve Thy1.1+ P14 cells were isolated from the spleen via magnetic bead (Miltenyi) sorting on Thy1.1 and labeled with 1uM carboxyfluorescein succinimidyl ester (CFSE) at 37°C for 15 min. 1 × 10^6^ CFSE-labeled naïve P14 cells were adoptively transferred i.v. into D-15 and D-6-infected mice. Three days post-transfer, spleens were harvested to monitor division of labeled P14 cells. Homeostatic proliferation (HP) assays were performed by injecting D99 memory sham and splx mice with 2 mg BrdU i.p. followed by 0.8 mg/ml BrdU in drinking H_2_O for 2 weeks. Bone marrow (BM), spleen, lung, and cervical, mediastinal, and inguinal lymph nodes were subsequently harvested, and P14 cells were analyzed for BrdU incorporation.

### Tissue preparation and flow cytometry

Single cell suspensions were prepared from BM, spleen, lung, and cervical, mediastinal, and inguinal lymph nodes at indicated times after transfer. Lungs were additionally incubated for 1 h at 37°C with DNAse (0.1 mg/mL) and collagenase (0.38 mg/mL) followed by a Percoll (Sigma; St. Louis, MO, USA) gradient isolation. Single-cell suspensions were diluted and counted on a hemocytometer using standard methods. Cells were labeled with anti-CD8, -CD62L, -CD27, -KLRG-1 (eBiosciences; San Diego, CA, USA), -Thy1.1, and -CD127 (Biolegend; San Diego, CA, USA) antibodies using standard manufacturer’s protocols. Cells were analyzed on an LSRFortessa flow cytometer (BD Biosciences; San Jose, CA, USA) and analyzed by Flowjo software (Tree Star; Ashland, OR, USA).

### Statistics

Unless indicated otherwise, significance was calculated by Student’s *t* test using Graphpad Prism 5 for Macintosh. *p*-values <0.05 were considered significant.

## Results

### Kinetics and representation of CD8 T-cells in the blood

**Figure 1:**
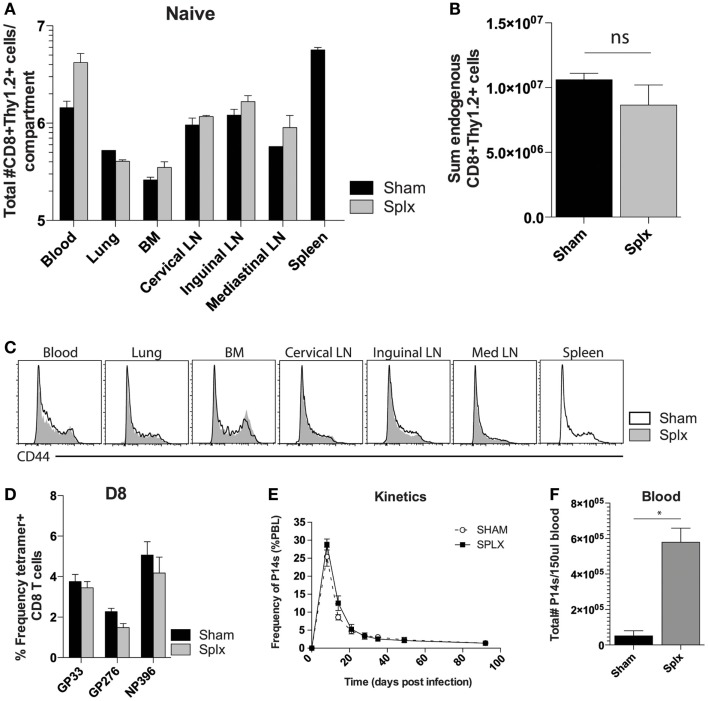
**Enhanced number of memory CD8 T-cells in the blood**. Naïve sham and splx B6 mice were sacrificed on the same day. **(A)** CD8+ Thy1.2+ cells were labeled and quantified in multiple tissue compartments. **(B)** Sum value of total number of CD8+ Thy1.2+ cells from all harvested organs. **(C)** CD44 expression on gated CD8+ Thy1.2+ population from harvested tissue. **(D)** Frequency of GP33+, GP276+, and NP396+ CD8 T-cells in the blood on D8 post LCMV-Armstrong infection. **(E)** 2 × 10^4^ naïve Thy1.1+ P14 cells were adoptively transferred into naïve sham or splx-treated mice (i.v.) and subsequently infected with 2 × 10^5^ PFU LCMV-Armstrong i.p. Frequency of P14 CD8 T-cells of total PBC was monitored over time. **(F)** At D100, numbers of P14 CD8 T-cells were quantified in 150 μl of blood. Data are represented as mean ± S.D. with five or more mice per group in three independent experiments. *p < 0.05.

Splenectomy has the potential to decrease the pre-immune repertoire of naïve CD8 T-cells. To address the impact of splenectomy on CD8 T-cell numbers, we quantified the CD8^+^Thy1.2^+^ T-cell compartment in multiple organs of naïve sham and splx C57BL/6 mice (Figure 1A). Numbers of CD8 T-cells were similar in lung, BM, and several lymph nodes. Of interest, we found higher numbers of CD8 T-cells in the blood of splx mice, which compensated from the lack of spleen, leading to an overall non-significant difference in the sum total of CD8 T-cells between sham and splx mice (Figure 1B). We further monitored CD44^hi^ and CD44^lo^ populations, with the result showing no organ specific or overall changes on the distribution of naïve and antigen-experienced CD8 T-cells in sham or splx hosts (Figure 1C). To address endogenous CD8 T-cell responses, we infected mice with the model viral pathogen, LCMV-Armstrong and analyzed tetramer positive CD8 T-cells, specific for three dominant epitopes (gp33-41, gp276-284, and np396-404) in the blood at day 8, the peak of the effector response (Figure 1D). We observed no significant difference between sham and splx mice in the frequency of LCMV-specific CD8 T-cells within the CD8 compartment. To continue studies of the effect of CD8 T-cell responses with a defined pre-immune repertoire, we adoptively transferred 2 × 10^4^ naïve tg P14 T-cells into sham and splx-treated B6 mice and infected them with LCMV, and performed longitudinal analyses in the blood. The frequency of total PBC and kinetics of the P14 response overlapped in both sham and splx mice (Figure 1E). Interestingly, the total number of lymphocytes (not shown) and tg P14 CD8 T-cells in the blood were approximately fivefold higher in memory splx compared to memory sham mice at >D100 (Figure 1F). Based on the higher number of lymphocytes in the blood of splx compared to sham mice, we investigated whether or not the well-described property of homeostatic proliferation (HP) of memory CD8 T-cells was altered long term in splx mice.

### Enhanced homeostatic proliferation of memory CD8 T-cells in splx, but not sham mice

**Figure 2:**
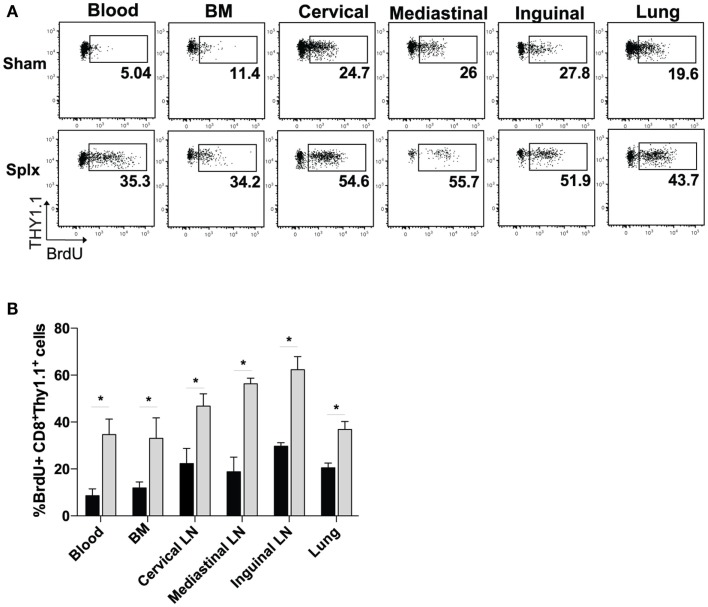
**Homeostatic proliferation of memory CD8 T-cells is enhanced in splx mice**. Memory P14 sham and splx mice were pulsed on BrdU for 2 weeks beginning on D99 post-infection. **(A)** Representative plots for BrdU incorporation of P14 cells in blood, BM, lung, and cervical, mediastinal, and inguinal lymph nodes at D106. **(B)** Summary bar graph from two independent experiments. Data are represented as mean ± S.D. with five or more mice per group. *p < 0.05.

Based on the higher circulating memory CD8 T-cell numbers in the blood 100 days post-infection, we hypothesized that HP of memory CD8 T-cells was enhanced by splenectomy. We pulsed memory sham and memory splx mice (D99) with BrdU for 2 weeks and harvested blood, BM, lung, and cervical, mediastinal, and inguinal lymph nodes. Memory P14 CD8 T-cells from splx mice had two- to sixfold higher frequency of BrdU positivity compared to memory P14 CD8 T-cells from sham mice (Figure 2A). HP of memory P14 CD8 T-cells was significantly greater (*p* < 0.05) in all tissues of splx, compared to sham, mice (Figure 2B). Our studies examining the overall CD8^+^Thy1.2^+^ T-cell population in naïve sham and splx mice (Figure 1A) suggest that splx mice are not lymphopenic. However, it was unknown whether the memory CD8 T-cell compartment experienced any defects or inflation in number. Hence, we next enumerated memory P14 CD8 T-cells from multiple tissues of sham and splx mice.

### Total number of memory CD8 T-cells remains intact in the absence of spleen

**Figure 3:**
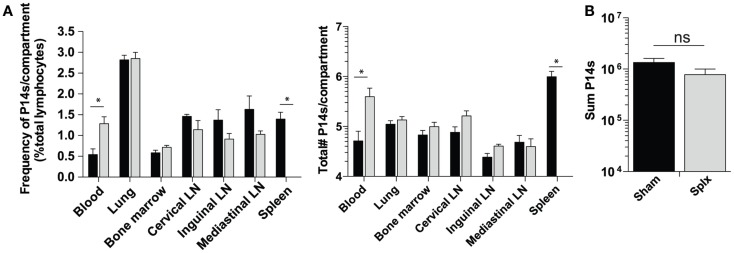
**Memory CD8 T-cell numbers equal in sham and splx mice**. Memory P14 sham and splx mice were sacrificed on D100. **(A)** Frequency and total number of P14 CD8 T-cells were quantified in multiple tissue compartments. **(B)** Sum value of total number of P14 CD8 T-cells from all harvested tissue compartments from two independent experiments. Data are represented as mean ± S.D. with five or more mice per group. *p < 0.05.

As our results show that HP of memory CD8 T-cells was substantially enhanced in splx hosts, we hypothesized that the number of memory P14 CD8 T-cells would be greater in each harvested tissue of splx, compared to sham mice, as we observed in the blood (Figure 1B). In contrast to this notion, frequencies (Figure 3A, left) and total numbers of memory P14 CD8 T-cells (Figure 3A, right) remained equal in every tissue, with the exception of the blood and spleen. Overall, the blood of memory splx mice contained roughly 10-fold higher numbers of memory CD8 T-cells compared to their sham counterparts (Figure 3A, right). The splenic compartment contained roughly 1 × 10^6^ memory CD8 T-cells in sham memory mice, which were absent in splx mice. Strikingly, despite the lack of the spleen, splx and sham mice had no significant difference in the total numbers of memory CD8 T-cells from all harvested compartments (Figure 3B). These data suggest that, despite enhanced HP of memory CD8 T-cells in splx mice, there are no overall increases in memory CD8 T-cell numbers in the host.

### Memory CD8 T-cell differentiation is similar in the absence of the spleen

**Figure 4:**
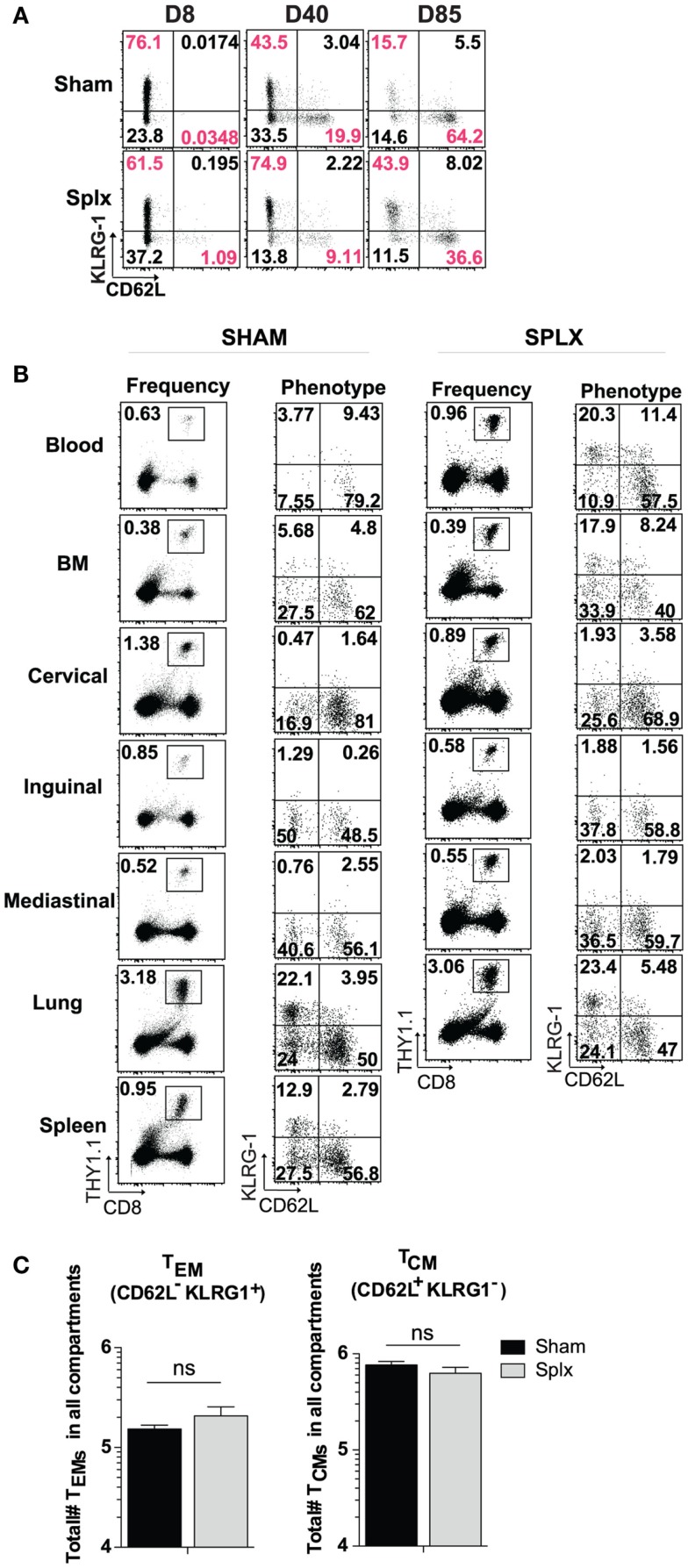
**Differentiation of memory CD8 T-cells in splx compared to sham mice**. 2 × 10^4^ naïve P14s were transferred into sham and splx mice, followed by 2 × 10^5^ PFU LCMV-Armstrong infection. **(A)** Representative KLRG1 and CD62L expression on P14 cells in blood at intermittent time points. **(B)** Representative phenotype of P14 cells in multiple tissue organs at D85 post-transfer. Representative plots shown. **(C)** Sum of total number of *T*_EM_ and *T*_CM_ P14 cells from all harvested tissue compartments. Data are represented as mean ± S.D. with five or more mice per group from three or more independent experiments. *p < 0.05.

In our longitudinal analysis of memory CD8 T-cell responses in the blood of sham and splx mice, we also evaluated the phenotype of memory CD8 T-cell subset markers in the blood. Across approximately 12 weeks, differentiation into the KLRG-1^lo^CD62L^hi^ central memory phenotype progressed as expected in sham mice. Interestingly, memory CD8 T-cells in the blood of splx mice retained a noticeable KLRG-1^hi^CD62L^lo^ population, indicative of effector memory phenotyping (Figure 4A). Based on these differences, we further analyzed memory CD8 T-cell phenotypes in multiple tissue compartments. Memory P14 CD8 T-cells exhibited little difference in KLRG-1 and CD62L phenotype with the exception of the blood (Figure 4B). As with total memory CD8 T-cell numbers, we additionally quantified the total number of effector (KLRG-1^hi^CD62L^lo^) and central (KLRG-1^lo^CD62L^hi^) memory CD8 T-cell numbers across multiple compartments. Interestingly, splenectomy did not affect the overall differentiation process of memory CD8 T-cells as total numbers of effector and central memory cells were similar (Figure 4C). Our data indicate that effector memory CD8 T-cells distribute to the blood in greater numbers in the absence of the spleen, but there is no general defect in memory CD8 T-cell differentiation in the splx host.

### Protective capacity of memory CD8 T-cells is unaltered despite lack of spleen

**Figure 5:**
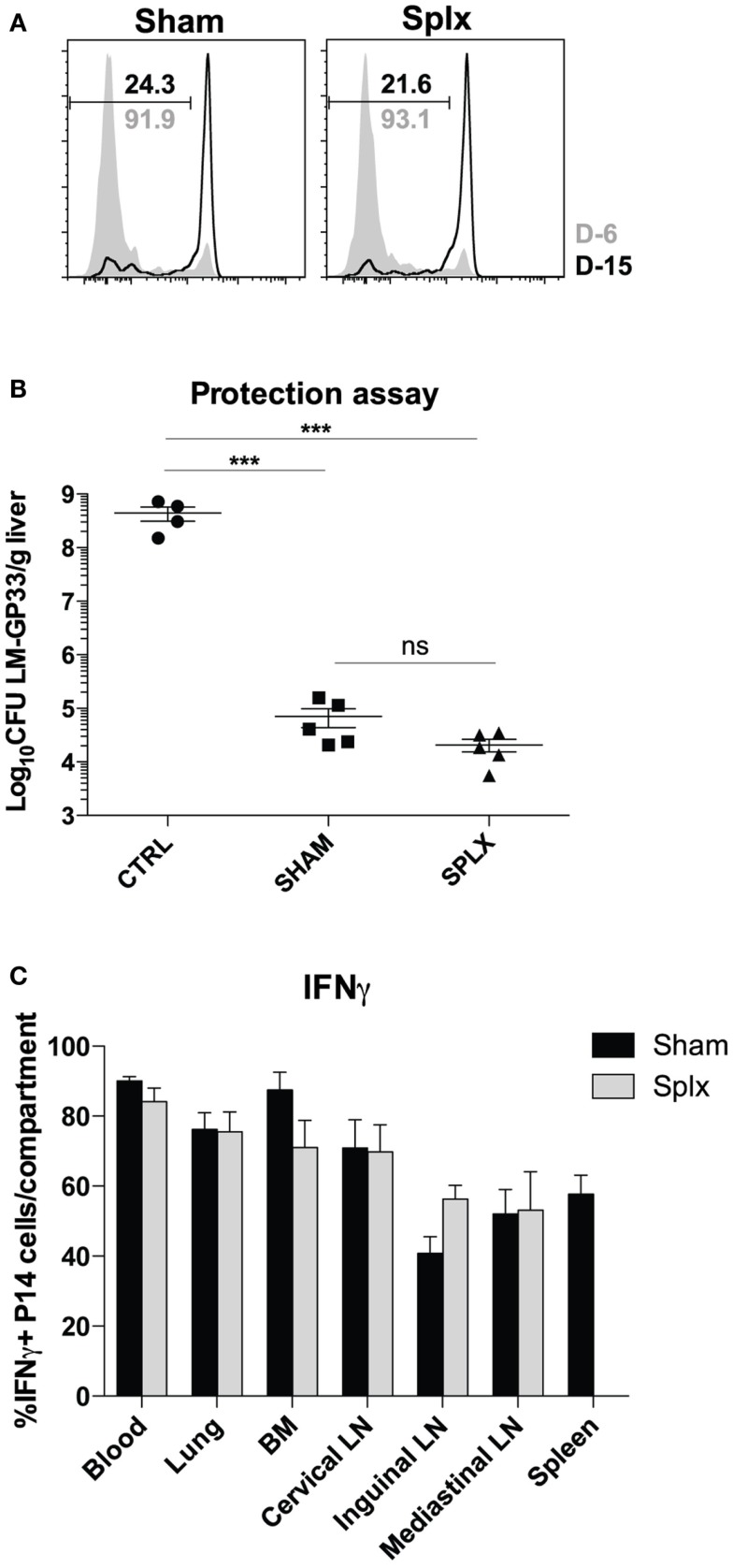
**Protective capacity of memory CD8 T-cells unaltered in the absence of spleen**. Sham and splx-treated mice were infected i.p. with 2 × 10^5^ PFU LCMV-Armstrong on D-6 and D-15. Naïve P14 CD8 T-cells were labeled with CFSE and adoptively transferred into infected mice at D0 (2 × 10^6^/mouse). Inguinal lymph nodes were harvested 3 days post-transfer and analyzed for CFSE dilution. **(A)** CFSE dilution of P14 cells from inguinal lymph nodes. **(B)** Naïve B6 mice and memory P14 sham and splx-treated mice were challenged with 1 × 10^6^ CFU virulent LM-gp33 (XFL203) intravenously. Bacterial burden in the liver was assessed on D3 post-challenge. **(C)** Frequency of IFNγ positive P14 cells in sham and splx mice. Data are represented as mean ± S.D. with five or more mice per group. ***p < 0.0005.

As numbers and subsets of memory CD8 T-cells are similar among sham and splx mice, we next investigated the functionality of these cells in either environment. First, we tested the ability of memory CD8 T-cells to clear LCMV-Armstrong in sham and splx mice. To do this, we infected sham and splx mice with LCMV-Armstrong at D-6 and D-15 with subsequent transfer of CFSE-labeled naïve CD8 T-cells on D0 to detect functional antigen display as a measure of antigen clearance. P14 CD8 T-cells from both groups of mice underwent similar division at D6 post-infection with essentially no division at D15 post-infection (Figure 5A). Thus, sham and splx mice had the same capacity to clear LCMV antigen. We next assessed protective capacity of memory CD8 T-cells in sham and splx mice via lethal infection with virulent *L. monocytogenes*. Naïve or memory P14 sham and splx mice were challenged with a high dose (1 × 10^6^ CFU) of virulent LM-gp33 intravenously. Both sham and splx mice reduced CFU of LM-gp33 in the liver 3 days post-infection by 4 logs compared to naïve controls (Figure 5B). Additionally, there was no significant difference in protective capacity between sham and splx mice. Finally, we assessed IFNγ production from memory CD8 T-cells in sham and splx mice. Across multiple tissue compartments, there was no significant difference in cytokine production between memory CD8 T-cells in a splx compared to sham host (Figure 5C).

## Discussion

The data presented here illustrate how HP and distribution of memory CD8 T-cells are altered in splx mice. We demonstrate that memory CD8 T-cells are represented in greater numbers in the blood of splx hosts. Our data show that memory CD8 T-cells undergo enhanced HP and that there are no defects in the protective capacity of memory CD8 T-cells in the absence of the spleen. Our comprehensive studies on CD8 T-cell responses generated in splx hosts support that splenectomy neither results in a lymphopenic environment nor vastly alters the distribution of memory CD8 T-cells. Thus, we speculate that the spleen may negatively regulate proliferation of T-cells, either directly or by the absence of tissue-resident cells that may regulate T-cell homeostasis (1). These data provide additional insight into the role of the spleen on various immune cells.

The splenic architecture, consisting of follicles with designated T-cell zones, suggests a prominent role for T-cell activation and protection from infection. Although there are some interesting changes that occur to the memory CD8 T-cell pool generated in a splx environment, CD8 T-cell kinetics and overall memory CD8 T-cell numbers remain similar in both sham and splx-treated mice. These findings are consistent with the clinical observation that the majority of splx patients are at higher risk for encapsulated-bacterial infections, rather than viral or intracellular bacterial infection. These data suggest that defects in memory CD8 T-cell function are not responsible for the greater risk in parasitic infections, such as malaria, which may be attributable to lower humoral immunity, lack of splenic CD4 T-cells (19), or failure to clear parasitized RBCs from lack of splenic architecture. These data further allow us to conclude that, despite its importance in germinal center reactions, the spleen plays a dispensable role in many aspects of CD8 T-cell biology. Our data do not preclude, however, that the spleen is necessary for CD4 T follicular helper cells, which may be a topic of future study.

We found the most interesting observation in our studies to be that long-term enhancement of HP of memory CD8 T-cells did not lead to greater memory numbers. These findings are supported by previous mechanistic studies in our lab that identify a T death intermediate memory (*T*_DIM_) cell subset generated via division of central memory CD8 T-cells to maintain stable numbers (20). It remains to be determined whether or not memory CD8 T-cells in a splx environment exhibit more cell death to balance their higher turnover. Conceptually, our studies suggest that the host is prone to limiting inflation of memory CD8 T-cell numbers in the absence of antigen, most likely to maintain repertoire diversity in the T-cell pool and prevent CTL-mediated autoimmune pathology.

## Conflict of Interest Statement

The authors declare that the research was conducted in the absence of any commercial or financial relationships that could be construed as a potential conflict of interest.
